# Effect of low-iron micronutrient powder (MNP) on the composition of gut microbiota of Bangladeshi children in a high-iron groundwater setting: a randomized controlled trial

**DOI:** 10.1007/s00394-021-02523-1

**Published:** 2021-02-25

**Authors:** Sabuktagin Rahman, Guus A. M. Kortman, Jos Boekhorst, Patricia Lee, Moududur R. Khan, Faruk Ahmed

**Affiliations:** 1 Public Health, School of Medicine, Griffith University, Gold Coast Campus, Parklands Dr., Gold Coast, QLD 4222 Australia; 2 NIZO Food Research B.V., Ede, The Netherlands; 3 Host-Microbe Interactomics Group, Wageningen University & Research, Wageningen, The Netherlands; 4 Institute of Nutrition and Food Science, University of Dhaka, Dhaka, 1000 Bangladesh

**Keywords:** Low-iron MNP, Gut microbiota, Groundwater iron, Bangladesh

## Abstract

**Purpose:**

Adverse effects of iron fortification/supplements such as Micronutrient Powder (MNP) on gut microbiota have previously been found in infection-prone African settings. This study examined the adversaries of a low-iron MNP compared with the standard MNP on the composition of gut microbiota in Bangladeshi children exposed to a high concentration of iron from potable groundwater.

**Methods:**

A randomized controlled trial was conducted in 2- to 5-year-old children, drinking groundwater with a high concentration of iron (≥ 2 mg/L). Children were randomized to receive one sachet per day of either standard MNP (12.5 mg iron) or low-iron MNP (5 mg iron), for 2 months. A sub-sample of 53 children was considered for paired assessment of the gut microbiome by 16S rRNA amplicon sequencing.

**Results:**

At baseline, the gut microbiota consisted of *Bifidobacteriaceae* (15.6%), *Prevotellaceae* (12.2%), *Lactobacillaceae* (3.6%), *Clostridiaceae* (4.1%) and *Enterobacteriaceae* (2.8%). Overall, there was no significant treatment effect of the low-iron MNP compared to the standard MNP. However, an apparent treatment effect was observed in children with a relative adult-like microbiota, with a higher relative abundance of potentially pathogenic *Enterobacteriaceae* after receiving the standard MNP compared to the low-iron MNP. This effect, however, was statistically non-significant (*p* = 0.07).

**Conclusion:**

In Bangladeshi children drinking iron-rich groundwater, a low-iron MNP supplementation did not have a significant impact on their gut microbiota profile/composition compared to the standard MNP.

The trial registration number is ISRCTN60058115; Date of registration 03/07/2019; retrospectively registered.

**Supplementary Information:**

The online version of this article (10.1007/s00394-021-02523-1) contains supplementary material, which is available to authorized users.

## Introduction

The Bangladeshi population is affected by a high burden of anemia. Two nationally representative surveys have reported the prevalence of anemia in 51% and 33% of preschool-age children [1–3]. However, contrary to the widely held assumption that iron deficiency (ID) is the most common cause of anemia, the prevalence of ID (10.7%) and iron deficiency anemia (IDA, 7.2%) was low [2]. A contemporary study in a north-western district of Bangladesh observed a zero prevalence of ID in women, while the prevalence of anemia was high (57%) [4]. These studies attributed the low prevalence of ID in the populations to the high level of iron in groundwater, which is the principal source of drinking water for the Bangladeshi population [3, 4].

Globally, iron supplementation and fortification programs have been recommended for the prevention and control of ID and anemia [5]. In-home fortification of micronutrients, where a caregiver adds vitamins and minerals to the weaning foods at home using micronutrient powders (MNPs) containing iron, has demonstrated a significant reduction in the risk of IDA in Bangladesh and other settings [6, 7]. The WHO recommended MNPs as an effective intervention to control IDA in children 6–23 months of age [8]. However, an excess of iron may lead to adverse effects. Recent fortification trials in Pakistan and Ghana showed that the use of MNP with 12.5 mg iron was associated with higher incidences of bloody diarrhea, respiratory infection [9] and hospitalizations attributed to diarrhea [10]. The findings of these studies generated discussion and commentary on MNP-induced adversaries. Iron is a growth-limiting nutrient for many gut bacteria; and as such these bacteria compete for unabsorbed colonic iron [11]. For most enteric Gram-negative bacteria (e.g., *Salmonella*, *Shigella* or pathogenic *Escherichia coli*), iron acquirement plays a significant role in expressing virulence and colonization [12]. In contrast, *Bifidobacteriaceae*,* Lactobacillaceae* and other beneficial bacteria in the colon provide an important ‘barrier effect’ against colonization and invasion by pathogens [13]. To complement this, recent studies in Africa have shown that MNP or iron fortification was associated with significant adverse influence in the intestinal microbial composition, leading to the proliferation of pathogenic bacteria (e.g., pathogenic *Enterobacteriaceae*) and a decrease in the number of health-promoting bacteria (*Lactobacillaceae*,* Bifidobacteriaceae*) [14–16]. Hence, a biological mechanism of iron-induced diarrhea as a result of iron supplementation or fortification has been established. Studies have yet to establish an optimum level of iron in supplements that would be efficacious in preventing anemia without increasing the risk of adverse effects. In African infection-prone settings, the use of MNP with 12.5 mg of iron in Kenyan infants induced an adverse effect on gut microbiota, i.e., an increase of potential pathogens such as *Enterobacteriaceae*, particularly *Escherichia/Shigella*, the *Enterobacteriaceae/Bifidobacteriaceae* ratio, and *Clostridium* [15]. The same study employing a low-iron MNP formulation containing 2.5 mg iron as NaFeEDTA failed to demonstrate an improvement of iron status. Moreover, compared to a placebo, it resulted in significant adverse changes in the gut microbiota [15]. Bangladesh, however, presents a different context. Since anemia in Bangladeshi children has been high [1, 2], the government has endorsed in-home fortification of MNP containing key micronutrients including iron (12.5 mg) in the national policy for the prevention of childhood anemia [17, 18]. For over a decade, the MNP program has been run by national Non-government organizations (NGOs). However, as stated above, the iron status in the populations is generally sufficient, and iron in the drinking groundwater plays a key role [2, 3]. This implies that iron deficiency has a modest role at best on the causation of anemia and there are other reasons for the condition in this setting, such as inadequate intake of other pertinent nutrients [3]. In the context where the population is exposed to a fair level of iron acquired naturally through drinking water, the existing programs of MNPs suffer from suboptimum coverage (2–3%, personal communication); and gastrointestinal side effects are reported to be important underlying factors [19]. In the Bangladeshi context of the high background level of groundwater iron, Rahman et al. examined the efficacy of an MNP with a low dose of iron (5 mg) in preventing childhood anemia against the standard MNP (12.5 mg iron), and assessed the comparative side effects. The results found a significantly lower incidence of side effects (e.g., diarrhea, nausea and fever) in the children who received the low-iron MNP [20]. To date, no study has been conducted to examine the effect of iron supplements on the gut microbiota in the Bangladeshi population. The present trial as a part of the Rahman et al. trial [20] examined the effect of an MNP with a low dose of iron (5 mg) compared with the standard MNP (12.5 mg iron) on the composition of the gut microbiota in Bangladeshi children exposed to a high concentration of iron from potable groundwater.

## Methodology

The study was conducted among children aged between 2 and 5 years, living in Belkuchi—a sub-district in north-western Bangladesh, approximately 125 km from the capital city, Dhaka. Belkuchi is an area where iron concentration in groundwater is predominantly high (≥ 2 mg/L) [21]. As ubiquitous in rural Bangladesh, people in Belkuchi rely on groundwater for the drinking purpose [21].

A total of 327 children were enrolled in the trial and were randomized to receive the standard MNP (containing 12.5 mg Fe as ferrous fumarate, 300 µg RE vitamin A, 5 mg zinc, 30 mg vitamin C, and 0.15 mg folic acid) and the low-iron MNP (identical except for 5 mg Fe as ferrous fumarate). The MNPs, manufactured by Manisha Pharmoplast Ltd, Gujarat, India, were packed in group-coded silver-colored identical sachets. Further information on randomization and group allocation is provided in Rahman et al. [20]. Children who took antibiotics within 2 months before enrollment and during the intervention period, children who took MNP/iron supplement within 2 months before enrolment, and children consuming MNPs below a specified amount (< 50 sachets) during the intervention were excluded from consideration in the study.

Written informed consent for the children’s participation was provided by their parents.

### Sample size, sampling and the procedure

**Fig. 1:**
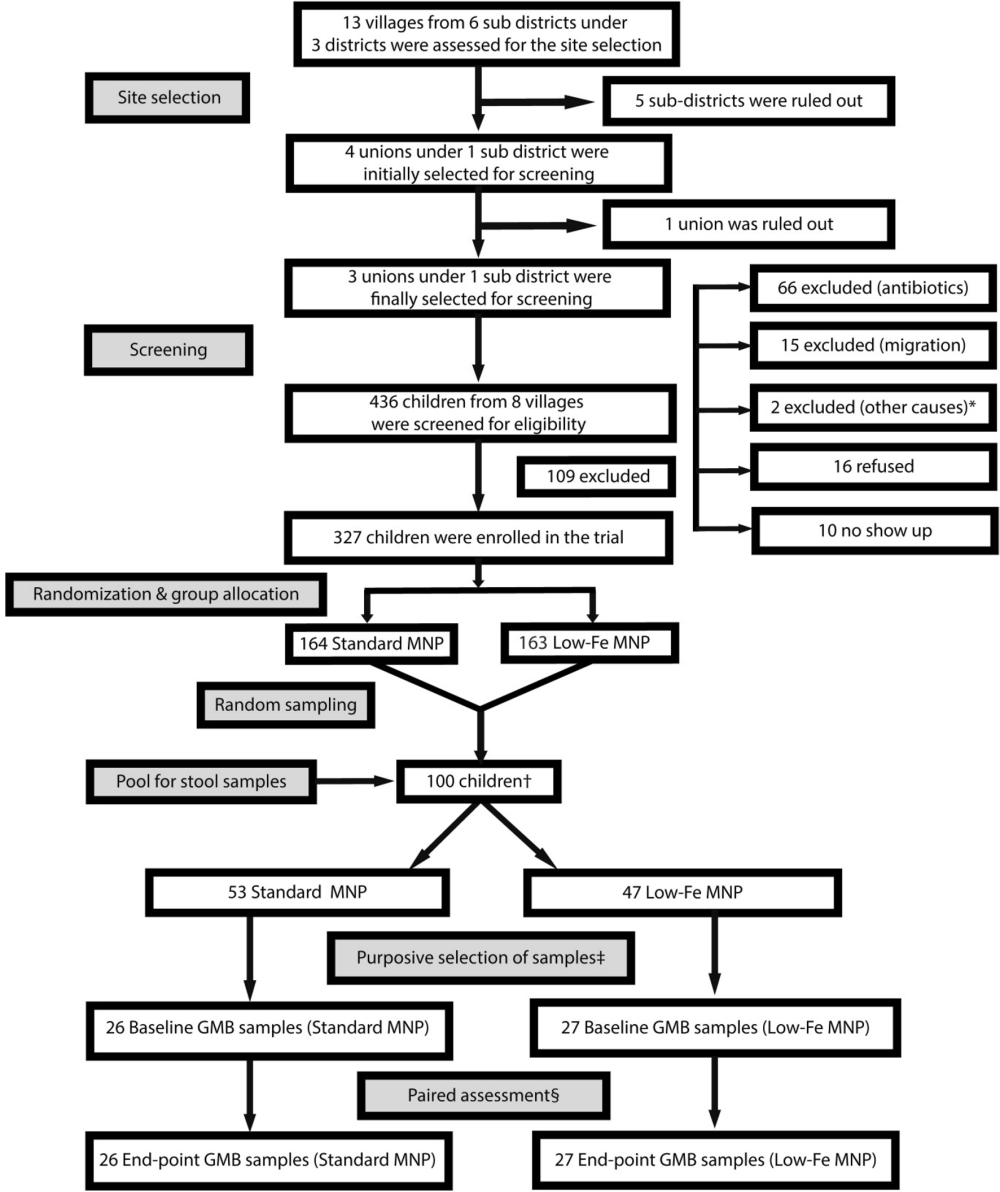
Selection of the children for gut microbiota assessment. ^*^Out of the stipulated age (*n* = 1); a tumor was found the abdomen (*n* = 1). †One hundred children were selected from the enrolled children (*n* = 327) randomly prior to the initiation of the MNP intervention as a pool to collect the stool samples from. This number was higher than the number required for the gut microbiota assessment. The priori consideration of higher number of the samples was done to buffer for the subsequent exclusion of cases in the cases of antibiotic would be taken by the children; and that they would consume MNPs at and above a specified level. ^‡^Purposive selection of samples was done after the endpoint data collection for the children not taking antibiotic during MNP intervention and consuming ≥ 50 sachets of MNP for microbiota assessment. ^§^Baseline samples are paired to the endpoint samples. *MNP* Micronutrient Powder, *GMB* Gut Microbiota

We considered 25–30 subjects per group would be adequate for comparison of the dominant bacteria based on previous studies [14, 22]. Hence, the required sample size was 50 for the two groups (standard MNP and the low-iron MNP). One hundred children were selected randomly from the enrolled children (*n* = 327) before the start of the intervention to form a pool of the stool sample for gut microbiota assessment. A higher number of stool samples than required (*n* = 50) was done to buffer for the subsequent exclusion of cases (children) who took antibiotics and/or consumed < 50 sachets of MNP over the 60-day intervention period. Fifty sachets, which translated to a compliance rate of ~ 84%, was determined based on a trial in Bangladeshi children that studied the efficacy and side effects of MNPs, reporting a ~ 85% adherence [7]. Following the intervention at the endpoint, it was found that 53 children had consumed >  = 50 MNP sachets (26 standard MNP and 27 low-iron MNP). The remaining 47 had either consumed antibiotics or had consumed fewer than 50 sachets, rendering them ineligible for gut microbiota assessment. The 53 children who had not taken antibiotics during the intervention period and had consumed >  = 50 sachets of MNPs were considered for gut microbiota assessment. Paired (baseline and endpoint) assessment of the samples resulted in a total of 106 stool samples being analyzed for gut microbiota (Fig. 1).

Before the intervention, mothers were shown how to mix rice with MNPs to feed to their children. Mothers were also instructed to feed their children one sachet of MNP every day for 60 days. During the 2-month intervention period, the children were visited each week by field personnel to record the occurrence of any illness including diarrhea, loose stools, nausea, vomiting, fever and acute respiratory infection over the preceding week. Compliance was assessed each week by the field personnel, who recorded the intake of the previous week’s MNPs by counting returned empty and intact sachets. During each visit, 10 sachets of MNP were provided to the mother to last until the next visit. Detailed procedures of the study are described elsewhere [20]. The high consumption (i.e., ≥ 50 sachets) of MNPs was considered for the analysis to enable that a fair amount of the iron supplement was consumed to induce an effect on the gut microbiota, since the duration of the intervention was relatively short, i.e., 2 months.

Iron concentration in the groundwater sample was measured using a Handheld Colorimeter (HI721 Checker^®^ HC (Hanna Instruments, USA), with a range between 0.0 and 5.0 ppm; a resolution of 0.01 ppm, and an accuracy ± 0.04 ppm ± 2% of the readings. Serum ferritin was measured by electrochemiluminescence immunoassay (ECLIA) on an automated immunoassay analyzer (Cobas C311; Roche Diagnostics, Mannheim, Germany), using a commercial kit according to the manufacturer’s instruction (Roche Diagnostics, GmbH, 68,305 Mannheim, Germany). Serum C-Reactive Protein (CRP) and 1-α Acetylated Glycoprotein (AGP) were determined by the particle enhance immunoturbidimetric assay on an automated, software-controlled clinical chemistry analyzer (Cobas c311, Roche Diagnostics GMBH, Mannheim 68,305 Germany) using commercial kits.

### Stool sample collection

The mothers of the selected children were briefed on how to collect stool samples, and were advised to have each child defecate on a square piece of paper that was provided for the purpose. Mothers were cautioned that the stool sample should not be mixed with urine. Immediately after the child had defecated, the mother folded the paper to cover the sample and then phoned a field attendant who collected the sample within 30–40 min. After discarding the top layer, the attendant used a sterile swab stick to collect ~ 5 g of the stool sample from the mid-layers of the mass in a sterile stool pot. The pot was capped and labelled with the sample ID and returned to the field laboratory in an ice box. The left-over stool sample was disposed of in the household’s toilet. At the field laboratory, the samples were refrigerated overnight at 3–4 °C, and dispatched in an ice-gel cool box to the laboratory in Dhaka early next morning. At the laboratory, the samples were homogenized on the same day, and aliquots were prepared with ~ 0.5 g of the homogenized sample in a cryovial, labelled and stored in a – 80 °C freezer. For DNA separation and sequencing, the homogenized stool samples were sent in dry ice to NIZO in the Netherlands.

### Bacterial DNA extraction, PCR amplification and 16S rRNA gene Illumina sequencing

Fecal samples were first thawed at 4 °C. Then, in a 2.0-mL screw-cap tube containing 0.5 g of 0.1-mm sterilized zirconia beads, 250 (± 10%) mg of feces and 700 µL S.T.A.R. buffer (Roche, Indianapolis, IN, USA) were added. The FastPrep instrument (MP Biomedicals, Santa Ana, CA, USA) was used for lysis at 5.5 ms for 3 times 1 min at room temperature. Thereafter, the samples were incubated while shaking at 100 rpm and 95 °C for 15 min. The samples were then centrifuged at 16,000×*g* for 5 min at 4 °C. The collected supernatant was kept on ice, and the lysis round was repeated once more as described above, except that only 350 µl S.T.A.R. buffer was added, with the remaining stool pellet. The supernatant kept on ice was then pooled with the supernatant from the second lysis round. Purification of DNA was performed on the automated Maxwell instrument (Promega, Madison, WI, USA) by applying the Maxwell 16 Tissue LEV Total RNA Purification Kit (Promega) according to the manufacturer’s instructions. To the first well of the Maxwell cartridge 250 µL of the supernatant was added and finally, DNA was eluted with 50 µL of RNAse/DNAse free water.

Using a 2-step PCR, barcoded amplicons from the V3–V4 region of 16S rRNA genes were generated (see library PCR below for a description of the second PCR step). For initial amplification of the V3–V4 part of the 16S rRNA universal primers with the following sequences were used: forward primer, ‘5-TCGTCGGCAGCGTCAGATGTGTATAAGAGACAGCCTACGGGAGGCAGCAG’ (broadly conserved bacterial primer 357F in bold and underlined); reverse primer, ‘5-GTCTCGTGGGCTCGGAGATGTGTATAAGAGACAGTACNVGGGTATCTAAKCC’ (broadly conserved bacterial primer 802R (with adaptations) in bold and underlined), appended with Illumina adaptor sequences (in italics). The PCR amplification mixture contained: 1 μl fecal sample DNA and 49 μl master mix (1 μl KOD Hot Start DNA Polymerase (1 U/μl; Novagen, Madison, WI, USA), 5 μl KOD-buffer (10 ×), 3 μl MgSO4 (25 mM), 5 μl dNTP mix (2 mM each)), 1 μl forward primer (10 µM), 1 μl reverse primer (10 μM) and 33 μl sterile water (total volume 50 μl). PCR conditions were: 95 °C for 2 min followed by 30 cycles of 95 °C for 20 s, 55 °C for 10 s, and 70 °C for 15 s. The approximately 500 bp PCR amplicons were then purified using the MSB Spin PCRapace kit (Invitek, Berlin, Germany). For the second PCR in combination with sample-specific barcoded primers, purified PCR products were shipped to BaseClear BV (Leiden, The Netherlands). PCR products were checked on a Bioanalyzer (Agilent) and quantified. This was followed by multiplexing, clustering and sequencing on an Illumina MiSeq with the paired-end (2×) 300 bp protocol and indexing. FASTQ read sequence files were generated using bcl2fastq2 version 2.18. Initial quality assessment was based on data passing the Illumina Chastity filtering. From the raw sequencing data, the sequence reads of too low quality (only “passing filter” reads were selected) were discarded and reads containing adaptor sequences or PhiX control were removed with an in-house filtering protocol. On the remaining reads, quality assessment was performed using the FASTQC tool version 0.11.5.

### 16S rRNA gene sequence analysis and statistics

16S rRNA gene sequences were analyzed using a workflow based on Qiime 1.8 [23]. We performed operational taxonomic unit (OTU) clustering (open reference), taxonomic assignment and reference alignment with the pick_open_reference_otus.py workflow script of Qiime, using uclust as clustering method (97% identity) and GreenGenes v13.8 as the reference database for taxonomic assignment. Reference-based chimera removal was done with Uchime [24]. The RDP classifier version 2.2 was performed for taxonomic classification [25]. Statistical tests were performed as implemented in SciPy (https://www.scipy.org/), downstream of the Qiime-based workflow.

### Statistical analysis

Characteristics of the participants, e.g., (age, ferritin status, CRP, AGP, and total intake of iron) and the concentration of iron in drinking water were presented as mean ± SD and median with interquartile ranges. The mean estimates were compared between the study groups by student’s *t* test. We tested for between-group differences in alpha-diversity (PD whole tree metric), phylogenetic distance (weighted UniFrac), and abundance of the taxa of primary interest (*Bifidobacterium*,* Enterobacteriaceae*,* Lactobacillus *and* Clostridiales*) without correction for multiple testing. In the bivariate explorative analysis of all taxa, the Mann–Whitney *U* test with FDR correction for multiple testing was applied to assess differences between the two groups. For comparisons of more than two groups, the non-parametric Kruskal–Wallis test with Dunn’s post hoc test was applied. For longitudinal analysis, the change of taxon relative abundance over time, 2log ratios were calculated, in which the relative abundance of a taxon at endpoint was divided by the relative abundance of the same taxon at baseline. Ratios were compared between groups by Mann–Whitney *U* tests with FDR correction for multiple testing.

We performed redundancy analyses (RDAs) on the gut microbiota composition as assessed by 16S rRNA gene sequencing in Canoco version 5.11 using default settings of the analysis type “Constrained” [26]. Relative abundance values of genera or OTUs were used as response data and metadata as the explanatory variable. For visualization purposes, families (and not OTUs) were plotted as supplementary variables. The microbiome age of a child at baseline was determined by RDA in which genera were response variables and calendar age was an explanatory variable. The x-coordinates of the cases (baseline samples) reflected the microbiome age (i.e., older children would have a more adult-like microbiota profile). The participants were divided by age group using the median value of the age distribution. The lower 50% of the cases were grouped in the “young” microbiome category and the higher 50% of the cases were grouped in the “older” microbiome category. Longitudinal effects of the intervention were assessed by calculating 2log ratios in which the relative abundance of an OTU or genus at endpoint was divided by the relative abundance of the same OTU or genus at baseline. These ratios were used as response variables in RDAs and were weighted based on the average relative abundance of each OTU in all infants. RDA calculates *p* values by permutating (Monte Carlo) the sample status. Partial RDA was employed to account for covariance attributable to age (always); age was first fitted in the regression modeling and then partialled out (removed) from the ordination as described in the Canoco 5 manual [26]. In all analyses, *p* values < 0.1 were considered modest statistical evidence [27]; *p* values < 0.05 were considered statistically significant.

Children’s age, biochemical and morbidity characteristics were compared between the groups by the independent sample *t* test.

## Results

At baseline, the treatment groups were similar with regard to the children’s age, mean concentrations of serum ferritin, CRP and iron concentration of groundwater (Table 1). Serum AGP at baseline was higher in the standard MNP group; 83.6 ± 31.0 mg/dL vs. 65.4 ± 17.1 mg/dL (*p* = 0.02), but most values were below the threshold of 100 mg/dL (values > 100 mg/dL indicate infection). The intake of iron from all sources (Fe from the diet, MNP and groundwater) over the intervention period was 20.9 ± 5.6 mg/d and 12.5 ± 5.1 mg/d in the standard and the low-iron MNP groups, respectively (*p* < 0.001, Table 1). The mean number of episodes of loose stool over the intervention period was 1.65 ± 5.7 and 1.48 ± 3.3, respectively (*p* = 0.89).

**Table 1:** Comparison of the groups with regard to children, biochemical characteristics, water iron concentration, total iron intake and morbidities between the treatment groups at baseline and over the intervention period

	Standard MNP (*n*, 26)		Low-iron MNP (*n*, 27)		*p* value**
	Mean ± SD	Median (IQR)	Mean ± SD	Median (IQR)	
Baseline					
Age (months)	42.8 ± 7.7		42.1 ± 7.8		0.74
Iron concentration in groundwater (mg/L)	14.9 ± 3.4	4.4 (3.7,12.6)	13.1 ± 3.1	4.2 (3.5,8.2)	0.07
Serum ferritin (ng/ml)	82.9 ± 36.9	81.9 (56.3,96.8)	71.4 ± 32.7	69.3 (54.2,94.6)	0.30
CRP (mg/L)	2.0 ± 2.6	1.0 (0.3,2.8)	1.9 ± 5.5	0.3 (0.3,0.5)	0.97
AGP (mg/dL)	83.6 ± 31.0	75.0 (62.0,74.0)	65.4 ± 17.1	62.5 (55.0,73.0)	0.02
Over the intervention					
Total Fe intake* (mg/day)	20.9 ± 5.6	18.7 (17.2,22.4)	12.5 ± 5.1	11.5 (9.5,14.6)	< 0.001
Mean episodes of loose stool	1.65 ± 5.7	0 (0,0)	1.48 ± 3.3	0 (0,0)	0.89

^*^Intake of iron from the combined sources of diet, groundwater and MNPs

^**^p values in relation to the mean difference

### General attributes of the microbiota samples

The average number of sequencing reads count per sample was 40,832. At baseline, there was a significant association between microbiota composition and calendar age (RDA; variation explained 1.3%, *p* = 0.05). Age was, therefore, considered as a covariate in subsequent multivariate analysis. Within-sample diversity, i.e., alpha-diversity, was not significantly different between the standard and the low-iron MNP groups at baseline and endpoint [Supplementary Fig. 1].

### Association of iron concentration of tube-well water with gut microbiota composition

**Fig. 2:**
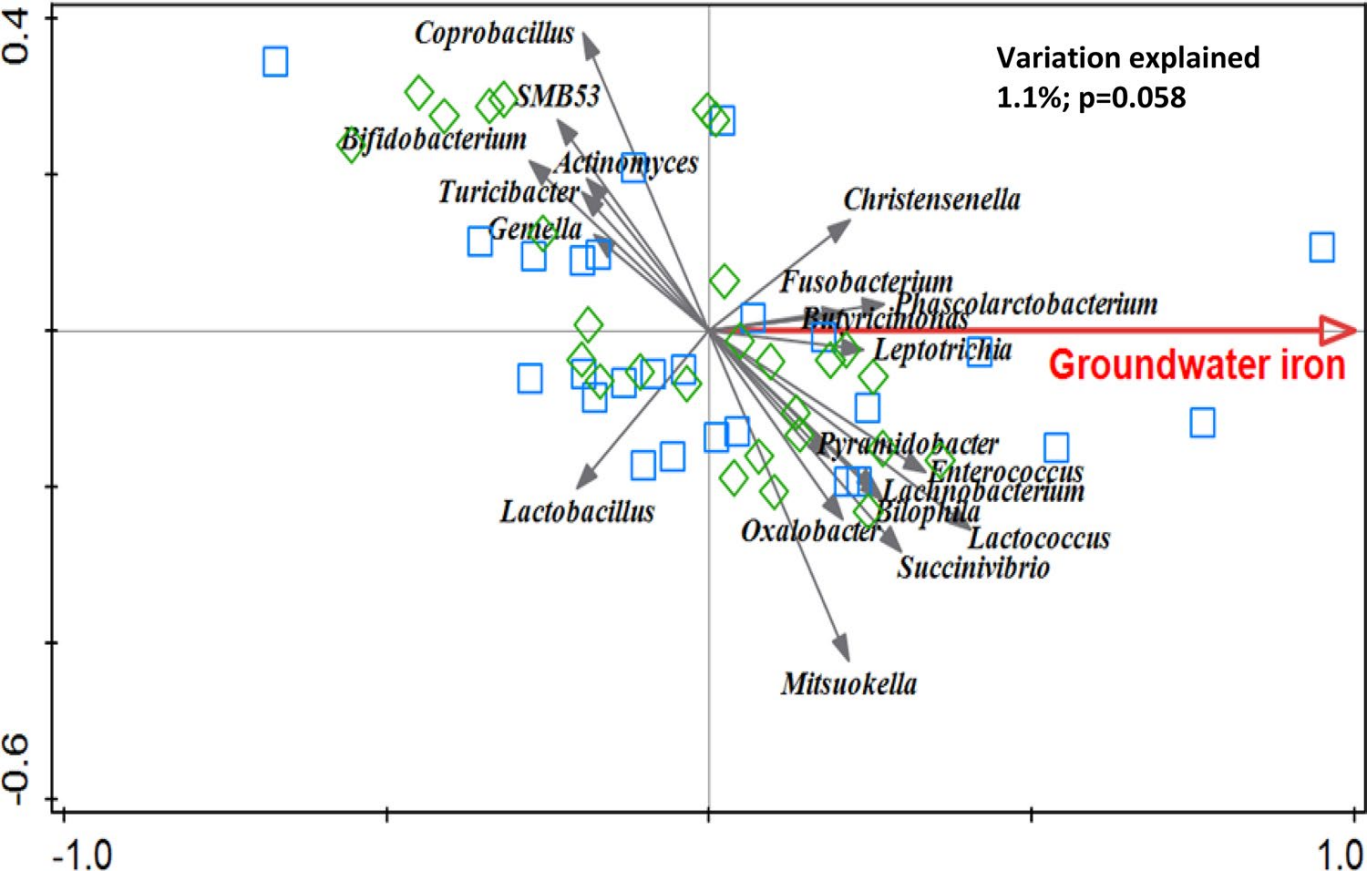
Redundancy analysis (RDA) on the genus level, assessing the effect of the concentration of iron in groundwater on gut microbiota composition at baseline. Genera were used as response data and groundwater iron concentration was explanatory data. Variation explained by groundwater iron concentration was 1.1%, *p* = 0.058. Blue squares indicate samples from the standard MNP group and green diamond samples represent children assigned to the low-iron MNP group

RDA at baseline showed that iron concentration of tube-well water (groundwater) was associated with the gut microbiota composition; variation explained 1.1% with a modest statistical evidence (*p* = 0.058, Fig. 2). *Bifidobacterium* and *Lactobacillus* were negatively associated with iron concentration.

### Baseline profile of gut microbiota and the overall treatment effect

**Fig. 3:**
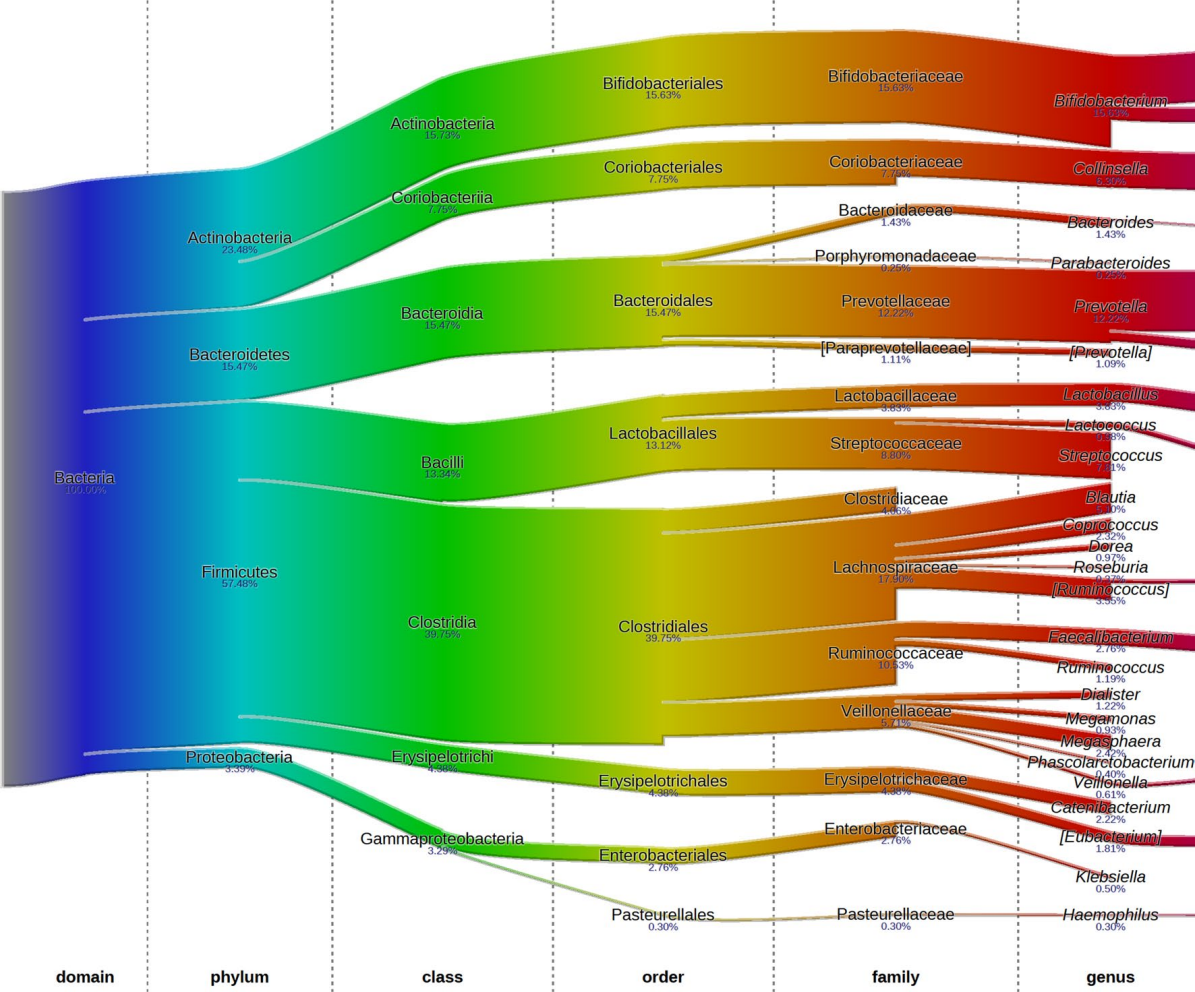
Average composition of the gut microbiota in Bangladesh rural children aged 2–5 years at baseline, exposed to a high-level iron acquired from drinking groundwater. The fraction of 16S rRNA reads (in %) attributed to specific taxonomic level is given below the taxon name. Figure was generated using software described in Sandquist et al. [28]

**Fig. 4:**
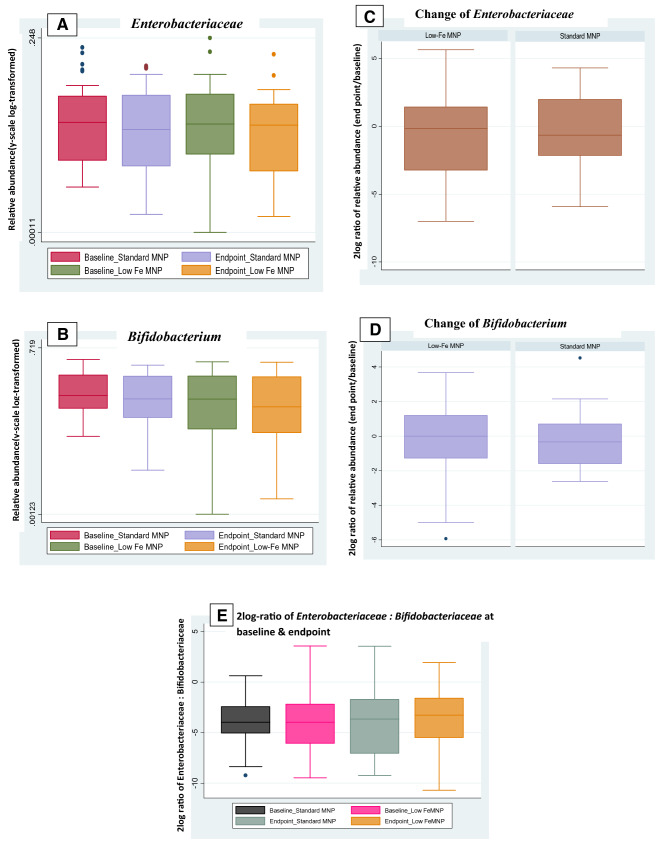
Relative abundance of **a** *Enterobacteriaceae*, **b** *Bifidobacterium* displaying the standard- and low-iron MNP groups over the baseline and endpoint time-points.[y-scale is log-transformed]. Boxplots of 2log ratios showing changes of relative abundances (from baseline to endpoint) of **c** *Enterobacteriaceae*, **d** *Bifidobacterium*, 0 = no difference between the time-points, 1 = twice as abundant at endpoint. Boxplots of 2log ratios showing, E. *Enterobacteriaceae*:* Bifidobacteriaceae at baseline and endpoint*

Average microbiota composition at baseline consisted of, among others: *Lachnospiraceae* 17.9%, *Bifidobacteriaceae* 15.6%, *Prevotellaceae* 12.2%, *Streptococcaceae* 8.8%, *Clostridiaceae* 4.1%, *Lactobacillaceae* 3.8%, and *Enterobacteriaceae* 2.8% (Fig. 3). There was no significant treatment effect on the overall microbiota composition as assessed by cross-sectional RDA and longitudinal RDA. Particularly, there was no effect on the relative abundance of *Enterobacteriaceae* and *Bifidobacteriaceae*. The relative abundance of *Enterobacteriaceae* at baseline, endpoint (Fig. 4a) and its relative changes over time (2log ratio) was not different between the treatment groups (Fig. 4c). Similarly, the relative abundance of *Bifidobacteriaceae* at baseline and endpoint (Fig. 4b) and its change over time (2log ratio) were not significantly different between the groups (Fig. 4d). Besides, there was no significant treatment effect on the 2log ratio of *Enterobacteriaceae*/*Bifidobacteriaceae* at endpoint (Fig. 4e). In the absence of a placebo group, microbiota composition was also compared between baseline and endpoint; there were no significant differences for the whole study population (both MNPs) and also not within the low- and standard-dose groups separately.

### Treatment effect on the gut microbiota at the subgroup level based on microbiome age

In the present study, the age of children varied between 24 and 59 months; the microbiota of younger children might respond differently compared to the microbiota of older children. However, RDA showed no difference in microbiota composition between the low-iron MNP and the standard-iron MNP at the endpoint in the younger age group or in the older age group (age groups were determined by dividing the children into two equal groups based on calendar age). As some younger children might have a relatively adult-like microbiota profile and some older children might have young microbiota, a microbiome age of each child was determined at baseline. Subsequently, children were categorized as either having a relatively young microbiota or a relatively old (adult-like) microbiota. RDA showed that MNP treatment was associated with gut microbiota composition in the old-microbiome group at the endpoint (variation explained 3.49%, *p* = 0.014) [Supplementary Fig. 3], but not in the young-microbiome group (0.0%, *p* > 0.05).

**Fig. 5:**
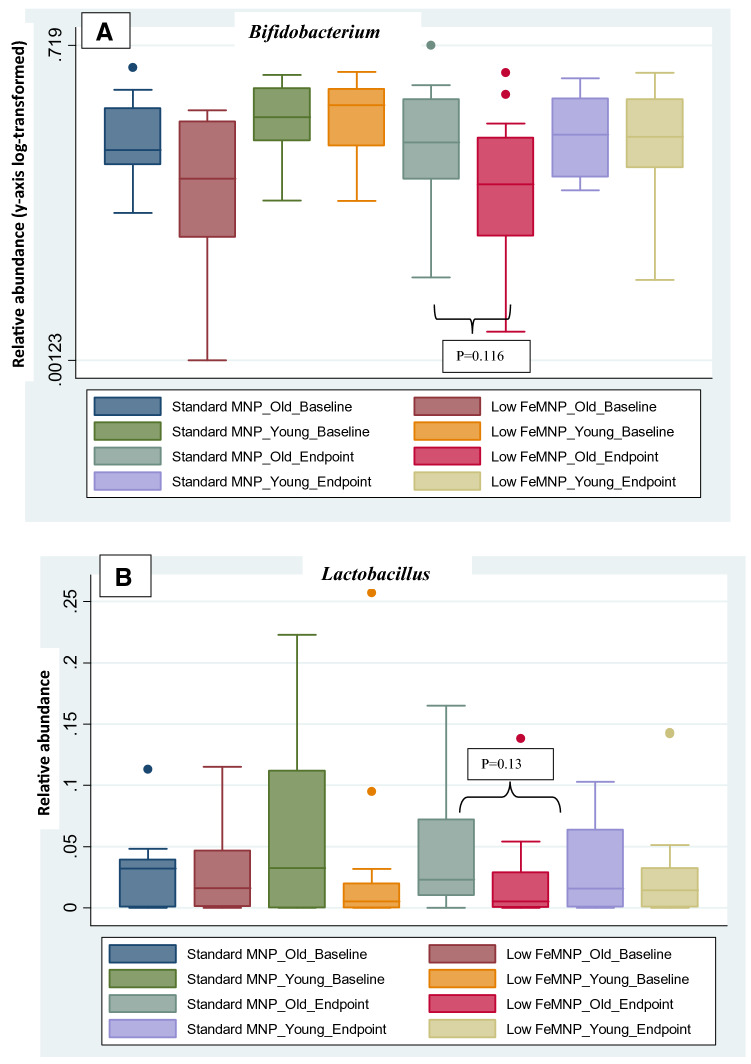
Relative abundance of **a** *Bifidobacteriaceae*, **b** *Lactobacillus* sorted by old- and young-age microbiota, comparing standard MNP and the low-iron MNP over baseline and endpoint time-points. Relative abundance was slightly higher at endpoint in standard MNP old-microbiome compared to low-iron MNP old-microbiome groups, both for *Bifidobacteriaceae* (*p* = 0.116) and *Lactobacillus* (*p* = 0.13); *but statistically non-significant*

**Fig. 6:**
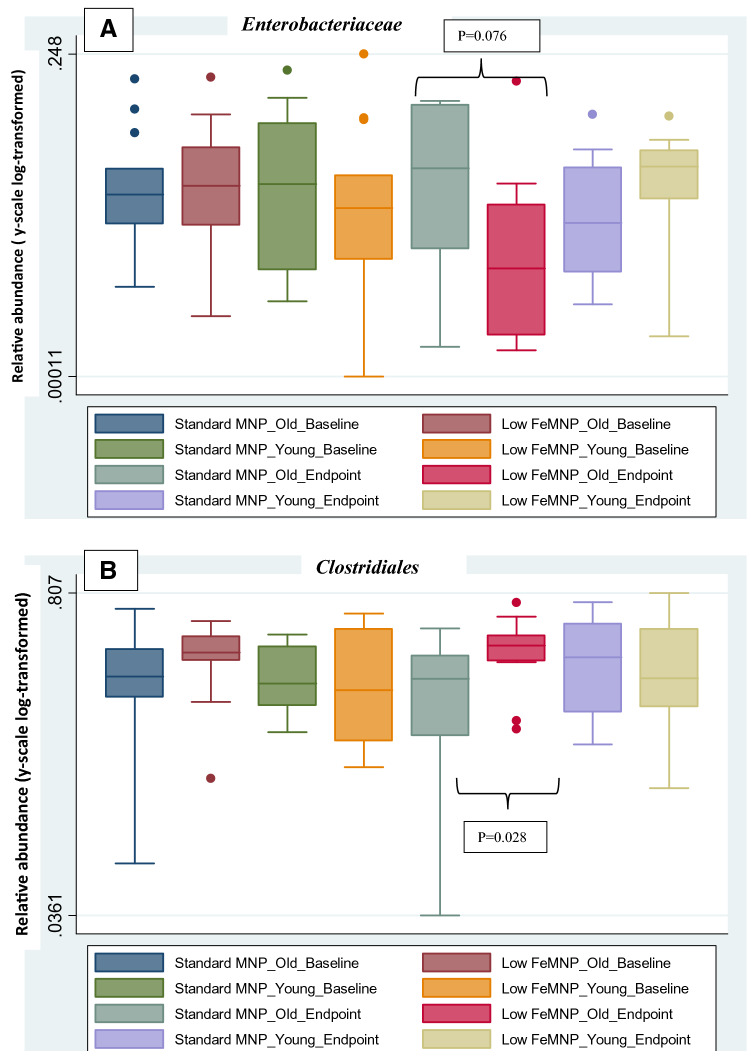
Relative abundance of **a** *Enterobacteriaceae*, **b** *Clostridiales* sorted by old- and young-age microbiota, comparing standard MNP and the low-iron MNP over baseline and endpoint time-points. The relative abundance of *Enterobacteriaceae* was slightly higher at endpoint in the sta*n*dard MNP-Old microbiome age group than in the low-iron MNP old-microbiome groups (*p* = 0.076). Low-iron MNP-old microbiome age group had higher relative abundance of *Clostridiales* at endpoint than in the standard MNP old-microbiome age group (*p* = 0.028)

Using bivariate analysis on the old-microbiome group, the relative abundance of *Bifidobacterium* and *Lactobacillus* in the standard MNP group at endpoint appeared to be higher compared with the low-iron MNP group; however, the difference was not statistically significant (*Bifidobacterium*, *p* = 0.116; *Lactobacillus*, *p* = 0.13) (Fig. 5). The relative abundance of *Enterobacteriaceae* at endpoint appeared higher in the standard MNP group compared to the low-iron MNP group [*p* = 0.076, (Fig. 6a)]. The relative abundance of *Clostridiales* was higher at endpoint in low-iron MNP group compared to the standard MNP group [*p* = 0.028, (Fig. 6b)]. In contrast, within the young microbiota group, there were no statistically significant treatment effects on these taxa of primary interest.

**Fig. 7:**
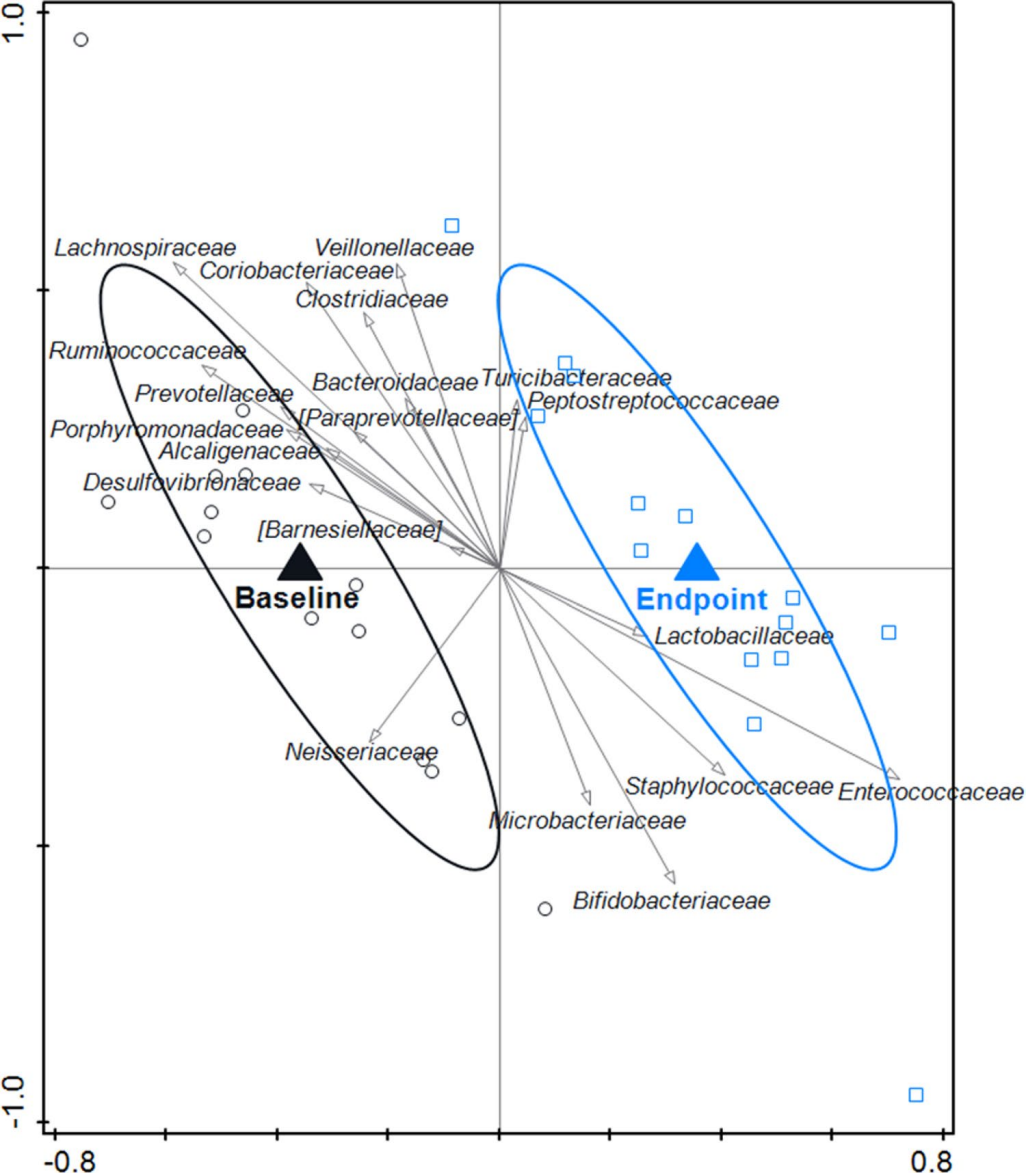
RDA on the OTU level, assessing the within-group effect of the standard MNP on the gut microbiota composition in the old-microbiome-standard MNP group. OTUs were used as response data and time point was explanatory data, the bacterial families that contributed most were plotted supplementary. The covariance attributable to subject was first fitted by regression and then partialled out (removed) from the ordination. Variation explained by time point was 2.6%, *p* = 0.088. The endpoint was associated with *Bifidobacteriaceae* and *Lactobacillaceae*

Using RDA, microbiota composition between baseline and endpoint was compared within the old-microbiota-standard MNP group, which showed modest evidence of statistical difference; variation explained 2.63% (*p* = 0.088). The endpoint was associated with *Bifidobacteriaceae*, *Lactobacillaceae* and *Enterobacteriaceae* (Fig. 7).

## Association of gut microbiota with iron status and infection biomarkers in children

There was no association observed between the iron status markers (hemoglobin and ferritin) and the composition of the microbiota at baseline and endpoint, as analyzed by RDA. At baseline, CRP, a biomarker for acute infection/inflammation, was associated with the gut microbiota composition; variation explained 1.88% (*p* = 0.04). CRP was associated with, e.g., *Peptostreptococcaceae* (a family that includes *Clostridium difficile* [29] but not with *Enterobacteriaceae*). Similarly, AGP, a biomarker for chronic infection/inflammation, was associated with the baseline gut microbiota composition with modest statistical evidence; variation explained 1.09% (*p* = 0.07) [Supplementary Fig. 2].

## Discussion

The present study explored the effect of a low-iron MNP compared to the standard MNP on the gut microbiota composition of Bangladeshi children exposed to a high amount of natural iron acquired from drinking groundwater. The iron doses in the low-iron MNP and the standard MNP were 5 mg and 12.5 mg per sachet, respectively, and were consumed by the children at one sachet per day for 2 months. The children of both groups consumed 84–100% of the doses (data not shown). Mean concentration of iron in groundwater was ~ 13 mg/L, which was several-fold higher than the cutoff (2 mg/L) [30].

In the present study, the baseline relative abundance of *Bifidobacteriaceae* was 15.6%, which was much lower than that reported in the studies of Kenyan infants (63–65%) conducted by Jaeggi et. al. [15] and Paganini et. al. [16]. The main reason for the difference is probably the age of the studied populations. The participants were infants in the African studies [15, 16] while the mean age ± SD of the present study participants were 43.5 ± 7.7 months. During infancy, the preponderance of *Bifidobacteriaceae* is linked with breast milk [31], which is the predominant form of food. The observation of a reasonable presence of *Prevotella* (12.2%) in the present study can be explained by the predominantly cereal-based, fiber-rich diet [32] which is the dietary characteristic in this setting. *Prevotella* is known for polysaccharide hydrolysis of the fibrous residue in the intestines. The baseline level of *Enterobacteriaceae* (2.8%) was similar to the results of the African studies (2.40–3.54%) [15, 16].

At baseline, the concentration of iron in groundwater was negatively associated with the relative abundance of the generally beneficial microbiota members *Bifidobacterium* and *Lactobacillus*. Drinking groundwater with a high concentration of iron is expected to increase the amount of iron in the intestines. Therefore, this observation is consistent with earlier studies [14–16] as iron tends to suppress the growth of these beneficial bacteria. Following the intervention, the combined intake of iron from MNP and groundwater at endpoint was not associated with the microbiota composition. A possible explanation for this is the fact that, at the endpoint, the intestinal load of iron was increased from consumption of MNPs in both groups. As a result, the association of a high load of iron in the intestine and the lower relative abundance of *Lactobacillus* and *Bifidobacterium*, at baseline, was attenuated at endpoint.

Our study showed: (A) no overall treatment effects on gut microbiota composition; and (B) some treatment effects on the old-microbiome subgroup, i.e., an apparent higher relative abundance of *Bifidobacterium* and *Lactobacillus* in the standard MNP group compared to the low-iron MNP group was observed. However, the difference was not statistically significant. For the potentially pathogenic *Enterobacteriaceae*, seeming modestly higher relative abundance (*p* = 0.076) was observed in the standard MNP, which is consistent with the Kenyan and the Côte d'Ivoire studies [3–5] as the high level of iron flared up the pathogens. The apparent higher relative abundance of *Lactobacillus* and *Bifidobacterium* in the higher iron group was unexpected. The trend of a higher relative abundance of *Enterobacteriaceae* in the old-microbiome-standard MNP group in combination with slightly higher relative abundances of *Bifidobacterium* and *Lactobacillus* is not considered a clear adverse effect of the standard MNP. The observation of seemingly modest treatment effects in the old-microbiome group only is difficult to explain, warranting further research. The apparent effect on *Enterobacteriaceae* in the small old-microbiome group is supported by the significant RDA on the old-microbiome group (*p* = 0.014; 3.5% variation explained), while RDA on the young-microbiome group was not significant (*p* > 0.05; 0.0%).

Overall, we speculate that the older calendar age of the children compared to the Kenyan infants’ studies could be one of the reasons for not finding the effects of MNP iron on the microbiota composition of the present study population. In the African studies assessing the effect of iron-fortified food and/or iron-containing MNP supplementation on the gut microbiota in infants, the salient observation was that iron supplements at various doses (2.5–20 mg) resulted in a significantly higher abundance of the (potentially) pathogenic microbiota, e.g., *Escherichia/Shigella*,* Clostridium* and *Enterobacteriaceae*, and pathogenic *E. coli* compared to “no-iron” and/or placebo intervention [14–16]. These studies further documented that the supplementation led to a suppression of the beneficial bacteria *Bifidobacterium* and *Lactobacillus*. The present study differed from these African studies in many ways. Such as the effect of two different doses of iron in MNP (12.5 mg vs. 5 mg) on the composition of the gut microbiota was compared in children aged 2–5 years old. Second, there was no placebo group. Third, the subjects were exposed to a high level of iron from groundwater, the natural drinking source; and finally, children taking antibiotic medicines during the intervention were excluded from microbiota assessment. Hence, different outcomes on the microbiota composition in the present study can be acceptable.

Similar to our study, a 38-week South African study providing 200 mg iron per week to 6- to 11-year-old children residing in a relatively malaria-free zone did not find any treatment effect on gut microbiota [33]. However, there were crucial differences in the design of that study to ours. In the South African study, the subjects were iron deficient, and the intervention was compared to a placebo. In a state of iron deficiency and low-infection burden, the supplements perhaps were absorbed well (Ferritin increased by ~ threefold after the intervention). Hence, conceivably less iron might have remained unabsorbed in the intestines which could be consistent with the lack of iron-induced adverse effects on the gut microbiome composition. On the other hand, a plausible explanation for the absence of an overall treatment effect in the present study is the comparison of the two MNPs, both containing iron (albeit, in different doses) and the absence of a placebo group, together with chronic exposure to a high level of iron from drinking groundwater. For ethical reasons, the trial did not include a placebo group [20]. Of note, when we assessed the pre-post differences for both the MNP groups separately, no treatment effect on microbiota composition was observed.

As stated elsewhere, excess iron might affect the gut microbiota adversely, resulting in morbidities. Hence, we assessed clinical morbidities, such as the occurrence of loose stools, in the treatment groups. The mean number of loose stools in the standard MNP and the low-iron MNP groups were 1.65 ± 5.78 and 1.48 ± 3.33, respectively; the difference was not statistically significant.

Modest evidence of association of the infection biomarkers CRP and AGP with the composition of gut microbiota was observed at baseline, but not at endpoint. *Enterobacteriaceae* were expected to be positively associated with the infection biomarkers, but this was not substantiated by our analysis. The difference in AGP between groups at baseline is not believed to have influenced our findings. In both the groups, only a few subjects (*n* = 2 in the low-iron MNP and *n* = 3 in the standard MNP group) showed AGP values slightly above the threshold of 100 mg/dL that indicates the presence of an infection.

In the parent trial, the low-iron MNP was non-inferior on hemoglobin response compared with the standard MNP and resulted in significantly fewer incidences of some key clinical morbidities, e.g., diarrhea, nausea and fever [20]. However, the gut microbiota assessment did not show a significant treatment effect on the overall gut bacterial composition, while some effects were observed in a subset of the population. Taking all these findings into consideration exemplifies that, despite there being fewer clinical morbidities from the low-iron MNP than the standard MNP, no clear comparative adverse effect of the standard MNP on gut microbiota composition was found. It is uncertain whether further lowering of the dose of iron (< 5 mg) would result in a favorable influence on the gut microbiome in this setting, and whether the iron reduction might compromise the efficacy on hemoglobin outcome; this should be a subject of future research. Further research is also needed to document the effect of the 5 mg Fe MNP on the gut microbiome in children residing in predominantly low-groundwater-iron areas.

A limitation of the trial is that the final samples were selected purposively for microbiota assessment. As such, the generalizability of the finding is somewhat compromised. However, strengths of the study are that the purposive sampling enabled high compliance children to be selected, and antibiotic users excluded. The effect of the MNP treatments on the composition of the microbiota was, therefore, not influenced by the effect of antibiotic treatments. Of note, this selection might in part have contributed to the observed lack of effects on microbiota composition, as oral antibiotics have been described to modify the effect of iron-containing MNPs on the gut microbiota composition in infants [34].

In conclusion, in Bangladeshi children naturally iron-replete from drinking groundwater, there was no overall significant treatment effect of the low-iron MNP on gut microbiota composition compared with the standard MNP. However, in a subpopulation with relatively adult-like gut microbiota, a seemingly higher relative abundance of potentially pathogenic *Enterobacteriaceae* was observed in children who received the standard MNP. Although we do not consider this as a clear adverse effect, this finding indicates a need for further research into the response of child gut microbiota types to iron supplementation.

## Supplementary Information

Below is the link to the electronic supplementary material.

Electronic supplementary material 1 (DOCX 900 kb)

## Data Availability

Data are available subject to reasonable request.
