# Supplementary material for: Effect of low-iron micronutrient powder (MNP) on the composition of gut microbiota of Bangladeshi children in a high-iron groundwater setting: a randomized controlled trial
Source: Eur J Nutr. 2021 Feb 25;60(6):3423–36. doi: 10.1007/s00394-021-02523-1 (PMC8354964; doi:10.1007/s00394-021-02523-1)
Supplement: Supplementary file 1 — Electronic supplementary material 1 (DOCX 900 kb) [file 394_2021_2523_MOESM1_ESM.docx]

Supplementary Fig. 1: Alpha-diversity or within-sample diversity (PD whole tree index) of the microbiota per treatment group and time point.

Indifferent over standard- and low-iron MNP groups at both the time points.

1. **1.9% variation explained; p=0.04**

**B. 1.1% variation explained; p=0.078**

Supplementary Fig. 2: RDA on the OTU level, assessing the effect of CRP (Panel A) and AGP (Panel B). OTUs were used as response data and CRP or AGP was explanatory data, the bacterial families that contributed most were plotted supplementary. The covariance attributable to age was first fitted by regression and then partialled out (removed) from the ordination. Variation explained by CRP and AGP was 1.9% (p=0.04) and 1.1% (p=0.078), respectively. Effect of CRP and AGP on the relative abundance of microbiota corrected for age (baseline). Blue squares indicate samples from the standard MNP group and green diamond samples represent children assigned to the low-iron MNP group.

**Variation explained 3.5%;**

**p=0.014**

Supplementary Fig. 3: RDA on the OTU level, assessing the effect of treatment on the relative abundance of the gut microbiota composition within the “old- microbiome-age group” groups at endpoint (corrected for real age). OTUs were used as response data and treatment was explanatory data, the bacterial families that contributed most were plotted supplementary. The covariance attributable to calendar age was first fitted by regression and then partialled out (removed) from the ordination. Variation explained by treatment was 3.5% (p=0.014). Blue squares indicate samples from the standard MNP group and green diamond samples represent children assigned to the low-iron MNP group.
